# Effects of Dehydroepiandrosterone on In Vitro Fertilization Among Women Aging Over 35 Years and Normal Ovarian Reserve 

**Published:** 2018-09

**Authors:** Fatemeh Mostajeran, Hatav Tehrani, Elham Ghoreishi

**Affiliations:** Department of Obstetrics and Gynecology, School of Medicine, Isfahan University of Medical Sciences, Isfahan, Iran

**Keywords:** Dehydroepiandrosterone, IVF, Infertility

## Abstract

**Objective:** The fertility potential of women declines with aging and the likelihood of infertility and stillbirth increase. Treatment protocols involving dehydroepiandrosterone (DHEA) provide pathways on improving fertility and live birth rate. This randomized clinical trial aimed to evaluate the effect of DHEA on In Vitro Fertilization (IVF) outcomes in women over 35 years and normal ovarian reserve.

**Materials and methods:** One hundred and six consecutive women with advanced reproductive age undergoing IVF due to infertility were enrolled in the study. Participants in the intervention group received 75 mg/day of DHEA orally 8 weeks before starting the cycle of ovulation induction. Participants in placebo group received placebo tablets during the same period. After 8 weeks, routine procedure of IVF was initiated then Clinical pregnancy, Miscarriage, Endometrial thickness and Duration of stimulation were evaluated for all the participants in both groups.

**Results:** There is no significant difference between groups in terms of age and BMI. Mean endometrial thickness was significantly lower in DHEA group (9.63 ± 0.70vs.8.05 ± 0.70; p < 0.001) while Duration of stimulation was higher (8.98 ± 1.29vs.10.59 ± 1.43; p < 0.001). There was no significant difference between pregnancy rate, and miscarriage rate of the groups.

**Conclusion:** According to the result of this study, DHEA supplementation may improve IVF outcomes in infertile women. Although additional larger and placebo-controlled studies using different DHEA protocols are required to support our present findings.

## Introduction

Worldwide, postponement of childbearing in following of the new perspectives for women, e.g. higher education and effective profession, is one of the challenging changes in the communities. Nowadays, too many women are delaying attempting to conceive until their mid-30s or later (1). The fertility potential of women decline with aging, then the likelihood of infertility and stillbirth increase (2, 3). Many strategies were proposed to manage infertility for women older than 35 years (3, 4); Among them, treatment protocols involving dehydroepiandrosterone (DHEA) increasing fertility specialists’ attention to prevail patients’ fertility and improving the live birth rate (4). 

The serum DHEA, a steroid secreted from the zona reticularis of adrenal glands, ovarian theca cell and by peripheral conversion, decreases significantly with advancing age. DHEA is an essential pro-hormone in ovarian follicular steroidigenesis and it is used as an anabolic steroid by the athletes (4). While conventional in vitro fertilization (IVF) treatment could not overcome to the age-related factors led to infertility, reducing ovarian reserve is one of the most important factors determining the success of IVF outcome. Improving ovarian response and clinical pregnancy rate in women with poor ovarian reserve is reported by some studies for IVF followed by DHEA supplementary (5-8). 

To our knowledge there is no sufficient information and randomized clinical trials on the effect of DHEA in infertile women. In this randomized clinical trial, we aimed to evaluate effect of DHEA on IVF outcomes in women over 35 years of age and normal ovarian reserve.

## Materials and methods

***Study design and participants:*** This is a randomized, controlled, double blinded clinical trial to evaluate the effect of DHEA on IVF outcome. One hundred and six consecutive women with advanced reproductive age undergoing IVF due to infertility at the Isfahan infertility center and Shahid Beheshti infertility center affiliated with Isfahan University of Medical Sciences (IUMS) between December 2015 and February 2017 were enrolled in the study. Participant with age more than 35 years, normal weight (18 ≤ Body Mass Index ≤ 25) and normal ovarian reserve (Local standard: FSH < 10 IU/L; AMH 2.0-6.80 ng/ml; inhibin B > 45 pg/ml) were included in the study. Semen analysis and spermiocolture, as routine infertility evaluation, were normal in all patients’ sexual partners. We excluded participants with a history of ovarian/pelvic surgery, diagnosed metabolic disorders, chronic diseases, and receiving DHEA and contraceptive before enrollment. After a detailed explanation of the study aims, informed consent was obtained from all participants before enrollment. The study protocol was reviewed and approved by the ethics committee of IUMS with number 394715. 

***Treatment protocol and outcomes evaluation: ***Through a computer-assisted block randomization of size 4, women were allocated at random to 2 groups (9).Participants in the intervention group received 25 mg three times daily of DHEA (Puritant`pride, USA)orally 8 weeks before starting the cycle of ovulation induction. Participants in placebo group received tablets contained ineffective lactose and similar in shape, color, and taste as DHEA tablets. After 8 weeks, IVF procedure was initiated according to our standard long-stimulation protocol for all the participants (10). Briefly, pituitary suppression was achieved by administration of triptorelin acetate (Decapeptyl, Ferring) 0.1 mg/daily subcutaneously in the mid luteal phase of the cycle preceding the IVF procedure. When down-regulation was achieved (indicated by estradiol concentration < 40 pg/ml and absence of folliles> 10 mm diameter), recombinant human FSH (Gonal F,Merck serono SA) 300 IU/daily subcutaneously was administered. 3-5 day after administration of rhFSH, according to individual ovarian response, assessed by serum levelsof estradiol and sequential transvaginal ultrasonography for evaluation endometrial thickness and diameter of follicles; when the leading follicle was > 17-18 mm diameter, human chorionic gonadotropin (hCG, Pregnyl) 10000 IU subcutaneously was injected. Oocytes aspiration was performed 36 hours later. Cycle was canceled if the follicles remained < 10 mm after 14 days of stimulation. 3 days after oocyte recovery, the 2 best quality embryos were transferred. Progesterone ampoule 50 mg twice daily intramuscular was started on the day following oocyte aspiration and continued for an additional 10 weeks to support luteal phase in case of pregnancy. Pregnancy was diagnosed by measuring increasing serum levels of β-hCG 14 days after embryos transfer. Clinical pregnancy was established if a fetal heart beat was observed by transvaginal ultrasonography. After clinical pregnancy, patients followed until 20 weeks .At the end of the study period, a checklist containing pregnancy (yes, no), miscarriage (yes, no), and standard IVF parameters, such as duration of stimulation (days of rhFSH treatment) and endometrial thickness was completed for all of the participants.

***Statistical analysis:*** Statistical package for social sciences version 22 (SPSS Inc., Chicago, IL, USA) was used to perform statistical analysis. Quantitative and qualitative variables were described by mean ± standard deviation (SD) and frequency (percent), respectively. Chi-squared or Fisher exact tests,when appropriate, were used to compare qualitative variables between study groups. Normality of the quantitative variables was assessed by Kolmogorov–Smirnov test and Q-Q plot, an independent t test was used to compare the variables between the groups for normally distributed continuous data. A p value less than 0.05 was considered statistically significant.

**Figure 1:**
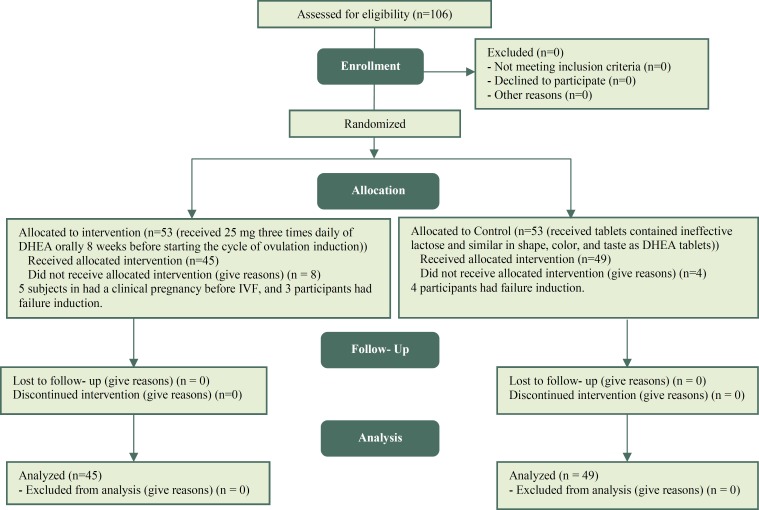
Flowchart Consort

## Results

One hundred and six participants who met the criteria for the study, 5 subjects in the DHEA group had a clinical pregnancy before IVF, and 3 participants in the DHEA group and 4 participants in the Placebo group had failure induction that were excluded from the analysis. Therefore, a total of 94 women (DHEA group: n = 45, Placebo group: n = 49) completed the study protocol (Figure 1).

**Figure 2:**
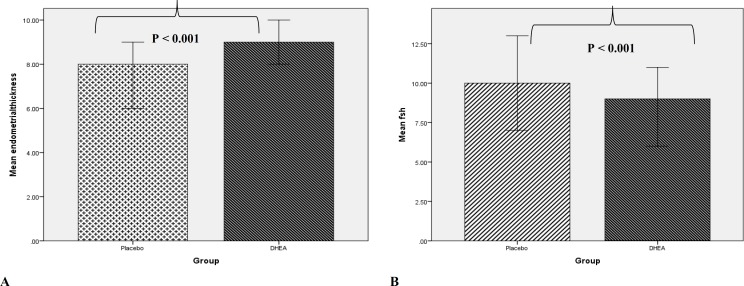
Comparison of endometrial thickness and duration of stimulation between two study interventions

Table 1 shows the results of comparison between the DHEA group and the placebo group. Two groups were similar in terms of Mean age and BMI (p value > 0.05). The clinical pregnancy rate in the DHEA group (46.7%) was higher than placebo group but with no significant different (36.7%). The miscarriage rate in the DHEA group (4.4%) was clinically lower than placebo groups (10.2%) but the difference was not statistically significant. Endometrial thickness was also significantly higher and duration of stimulation lower in DHEA group than placebo group and both were statistically significant (p value < 0.001) (Figure 2).

**Table 1:** Comparison of basic characteristics and clinical outcomes between the two study groups

	**DHEA** **n = 45**	**Placebo** **n = 49**	**p value** *
Age(years)	37.07 ± 1.19	37.24 ± 1.27	0.486
BMI(kg/m^2^)	22.31 ± 1.31	22.67 ± 1.09	0.156
Clinical pregnancy	21(46.7%)	18(36.7%)	0.329
Miscarriage	2(4.4%)	5(10.2%)	0.438
Injection failure	3(6.6%)	4(8.08)	0.51
Endometrial thickness(mm)	9.63 ± 0.70	8.05 ± 0.70	< 0.001
Duration of stimulation(day)	8.98 ± 1.29	10.59 ± 1.43	< 0.001

* Resulted from independent t-test for continuous and chi-square or Fisher exact test for categorical variables

## Discussion

Through a randomized double blinded, controlled trial, our study showed that DHEA could improve some IVF outcomes in women with advanced age and normal ovarian reserve.

One of the most important factors affects women’s fertilization is age. Due to the social trend of delay in childbearing, number of women with age related infertility and pregnancy loss is increasing. At birth, the ovaries contain 1-2 million primordial follicles and at menarche only 300000-400000 remain. During the reproductive years, the decline will continue and gradually accelerate that led to the numbers to drop below 1000 at the time of menopause. Along with the decrease in follicle number, oocyte quality also diminishes. It is believed that the loss of oocyte quality to be due to an increase in meiotic non disjunction resulting in an increasing rate of aneuploidy in the early embryo at female with older ages (1). Among the several treatment strategies, DHEA is proposed to overcome infertility and to improve IVF outcomes. Many studies have suggested that DHEA treatment may be effective in poor responders (7, 11-14). Some evidence suggests that androgens may be essential normal folliculogenesis and female fertility (6, 15).

In current study we observed that in the DHEA group, clinical pregnancy rate was higher and miscarriage rate was lower than the placebo group. Although these findings were not statistically significant, however the difference between two groups were in line with other previous studies clinically significant (2, 8). Tartagni et al (2) had found that higher, but not significant pregnancy rate in DHEA group, Also they reported significantly lower miscarriage rate in the DHEA group. Researchers’ hypothesized decreasing rate of miscarriage is related to a decreased aneuploidy rate. This hypothesis was demonstrated by Gleicher et al (6) that DHEA pretreatment is correlated with significantly reduced aneuploidy. Due to lacks of further exploration about the causes of miscarriages in our study we could not provide sufficient information to relate DHEA, aneuploidy, and miscarriage rate. Also, our results showed that endometrial thickness in the DHEA group was significantly higher than the placebo groups; and, duration of stimulation in the DHEA group was significantly lower than the placebo group. It suggests that exogenous DHEA increase the ovarian sensitivity to gonadotrophin stimulation in women with advanced reproductive age. These findings are in line with the other studies (16). Xuemei Liu et al reported duration of required FSH for ovarian stimulation was significantly decreased in the DHEA group but endometrial thickness was not significantly increased in the DHEA group (16).

The current study as the first report from Iran, was conducted in a formal framework of randomized controlled double blind clinical trial with, fairly proper sample size. The finding of our study is limited to the studied population and should be interpreted with cautions.

## Conclusion

Our results suggest that IVF with DHEA pretreatment has potential for improving fertility in women with advanced age and normal ovarian reserve. Although current study as the first randomized, double blind clinical trials in Iran suggested potential benefits of DHEA pretreatment there is a great need for conducting larger studies enjoying with strong methodological considerations in order to support our results. For better infertility management in women with advanced reproductive age and normal ovarian reserve.
